# Ability of neural network cells in learning teacher motivation scale and prediction of motivation with fuzzy logic system

**DOI:** 10.1038/s41598-021-89005-w

**Published:** 2021-05-06

**Authors:** Zahra Pourtousi, Sadaf Khalijian, Afsaneh Ghanizadeh, Meisam Babanezhad, Ali Taghvaie Nakhjiri, Azam Marjani, Saeed Shirazian

**Affiliations:** 1 Department of English, Science and Research Branch, Islamic Azad University, Tehran, Iran; 2 Department of Education, Faculty of Education and Psychology, Shahid Beheshti University, Tehran, Iran; 3 Imam Reza International University, Mashhad, Iran; 4 Institute of Research and Development, Duy Tan University, Da Nang, 550000 Vietnam; 5 Faculty of Electrical-Electronic Engineering, Duy Tan University, Da Nang, 550000 Vietnam; 6 Department of Artificial Intelligence, Shunderman Industrial Strategy Co., Tehran, Iran; 7 Department of Petroleum and Chemical Engineering, Science and Research Branch, Islamic Azad University, Tehran, Iran; 8 Department Chemistry, Arak Branch, Islamic Azad University, Arak, Iran; 9 Laboratory of Computational Modeling of Drugs, South Ural State University, 76 Lenin Prospekt, 454080 Chelyabinsk, Russia

**Keywords:** Mathematics and computing, Computational science, Software

## Abstract

We employed a new approach in the field of social sciences or psychological aspects of teaching besides using a very common software package that is Statistical Package for the Social Sciences (SPSS). Artificial intelligence (AI) is a new domain that the methods of its data analysis could provide the researchers with new insights for their research studies and more innovative ways to analyze their data or verify the data with this method. Also, a very significant element in teaching is teacher motivation that is the trigger that pushes the teachers forward, depending on some internal and external factors. In the current study, seven research questions were designed to explore different aspects of teacher motivation, and they were analyzed via SPSS. The current study also compared the results by using an adaptive neuro-fuzzy inference system (ANFIS). Due to the similarity of ANFIS to humans' brain intelligence, the results of the current study could be similar to humans regarding what happens in reality. To do so, the researchers used the validated teacher motivation scale (TMS) and asked participants to fill the questionnaire, and analyzed the results. When the inputs were added to the ANFIS system, the model indicated a high accuracy and prediction capability. The findings also illustrated the importance of the tuning model parameters for the ANFIS method to build up the AI model with a high repeatability level. The differences between the results and conclusions are discussed in detail in the article.

## Introduction

As mentioned by Gardner in 1985, motivation has been recognized as the essential item in the teaching and learning context, especially in second language learning; moreover, this factor is significantly influential as well. Therefore, it means that the learners need the motivation to pursue their studies^1^. Studies revealed that motivation could have two different internal or external sources, and teachers are the main external source of their learners' motivation^2^, which is in harmony with Gardner et al., meaning that teacher motivation is significant due to its impact on student motivation^3^. These studies also in line with Dörnyei, whose study shows that teacher motivation could change student learning achievements as well as student motivation^4^.

Teacher motivation matters because the future of every society connects to their teachers and how they educate and prepare students in the classes for their future lives and professions. So by investigating teaching motivators, useful information can be obtained for a more productive class. Therefore, there are some studies focusing on this topic.

A qualitative research study in 2018 focused on determinants and consequences of teacher motivation and demotivation^2^. This research was the backbone of the researchers’ next study. Therefore, Pourtousi and Ghanizadeh^5^ designed and validated a scale for measuring teacher motivation based on the collected data from the triangulated qualitative study that was proposed by Pourtoussi et al.^2^. The researchers designed a questionnaire based on five factors that is EFL teachers’ motivation scale (TMS) in order to measure teacher motivation. Different factors affect teachers, and the researchers of the mentioned study considered them in designing the TMS.

Moreover, a research study that included a variety of analyses indicated that teachers and their emotional support found that teacher support can greatly affect the understanding of the students regarding the instructional and emotional support from their teachers, leading to students’ harder work in the class context^6^. Based on another research, teacher motivation is one of the most crucial elements that greatly affect teaching and its quality^7^. Thoonen et al. revealed in their paper that various characteristics of teachers, which include their gender and teaching experience, have their own role in their motivation, and they can easily affect their motivation. The researchers of the same study understood that teacher experience in elementary education influences teacher engagement in a positive way. This implies that more experienced elementary teachers are much more willing to be up to date compared to those who are less experienced. Also, more experienced teachers are much more willing to internalize and follow the goals of schools. However, the researchers found out that the experienced teachers have less tendency in order to participate in different kinds of activities leading them to uncertainty. The same study also elucidates the gender differences in teaching, which is the result of the engagement of teachers in the research experiment. Work motivation and job satisfaction are two important factors among teachers that Arifin emphasizes, and the researcher of the study shed light on the positive influence of motivation on teacher performance and how organizational culture could affect teacher performance^8^. Research studies also focus on the positive influence on complex relations of motivation between the two processes of teaching as well as learning^9^. Kocabas explained in the research paper that the origins of motivation differ, which is because of two reasons^10^. The first reason is that people are different due to their different desires, needs, values, attitudes, and expectations. And the second reason is that humans are social and psychological creatures. The mentioned two reasons lead to this conclusion that the researchers could not have definite origins for teachers in order to motivate them at the same level. Another research study provided some other elements that can influence teacher motivation. The factors are income status, importance in society, self-confidence, rewards for showing good results^11^. Also, studies show that a higher level of autonomous motivation and a low level of controlled motivation can lead to a higher level of intrinsic motivation as well as self-determination^12^. According to the study, social-contextual conditions can affect the teachers. The study reveals that teachers have three categories of pressure at work. The first one originates from the school environment, which consists of teachers' perception referring to the responsibility of teachers for the students' behaviors and performance. The second one relates to the perception of teachers toward teaching methods. And finally, the last one refers to the teacher's perception of the limitations of their freedom in the curriculum. Another relevant study in 2015 proposed the two different leading to teacher motivation and helping to the recruitment include policymakers and teacher educators. The study also indicates that contextual and individual factors can change the amount of teacher motivation. Heinz clarified effect of policymakers and teacher educators on teacher motivation. So the contextual and individual factors have the possibility to have their own impact on motivation, which are teacher's tasks, teacher's responsibilities, environment and conditions of the workplace, job security, payment, cultural sides, social sides, each teacher's socio-demographic backgrounds, prior education, and professional opportunities^13^.

These days, the classes may become online using Artificial intelligence (AI) methods, since the coronavirus infects numerous numbers of people, and as far as studies have started to focus on the online classes during the COVID-19 pandemic^14–20^, the role of the teachers and their motivation besides the AI become very significant. AI has a significant influence on our everyday life^21^, and it can lead to the economic development of the countries. This technology has been developed, but some limitations exist; therefore, some researchers proposed their intelligent model that can create new ideas without the need to have similar experiences called brain intelligence (BI)^21^.

Moreover, AI could be mixed with speech recognition technology in digital assistants to provide customers' services based on their preferences^22^. Some instances of the assistants include Google Assistant, Siri, and Alexa, which are designed for Google, Apple, and Amazon, successively. These assistants can provide a variety of tasks for their users^23^. AI, or specifically machine learning, has its own role in most of the fields. The most well-known and typical example of using machine learning is its use in games. In the game industry, AI methods are also applied, including neural networks, fuzzy logic, genetic algorithms, and so on^24^. Machine learning can also be used for detecting as well as supervising phishing websites while people are using online services by using the algorithms^25^.

AI techniques have widespread applications in medical domains, and according to Jiang et al.^26^, some of the methods are more popular, including neural networks and support vector machine (SVM). Based on the same research study, neurology, cardiology, as well as cancer are three main diseases that AI can be used for them. Moreover, Jiang et al. indicated this technology could be used for different stages of stroke from early diagnosis to prognostications. Furthermore, AI could be advantageous for psychologists as well as professionals in the health care domain to help patients^27^.

ANFIS stands for adaptive neuro-fuzzy inference system that is an artificial neural network on the structure of the fuzzy framework and a mathematical tool in order to explain a system that was proposed by Takagi and Sugeno^28^. Some studies proved that using soft computing methods is possible in a variety of studies. A researcher in Iran used the support vector machine in order to predict anger expression with a suitable accuracy^29^. Another study in Japan used the integration of structural equation modeling (SEM) as well as the ANFIS algorithm for evaluating the safety paradigm in the petroleum-based sector^30^. Moreover, according to an investigation in southeastern Poland, the researchers applied Partial Least Squares Structural Equation estimation toolbox and ANFIS for modeling components of their survey relating to employees’ safety at work^31^.

**Figure 1:**
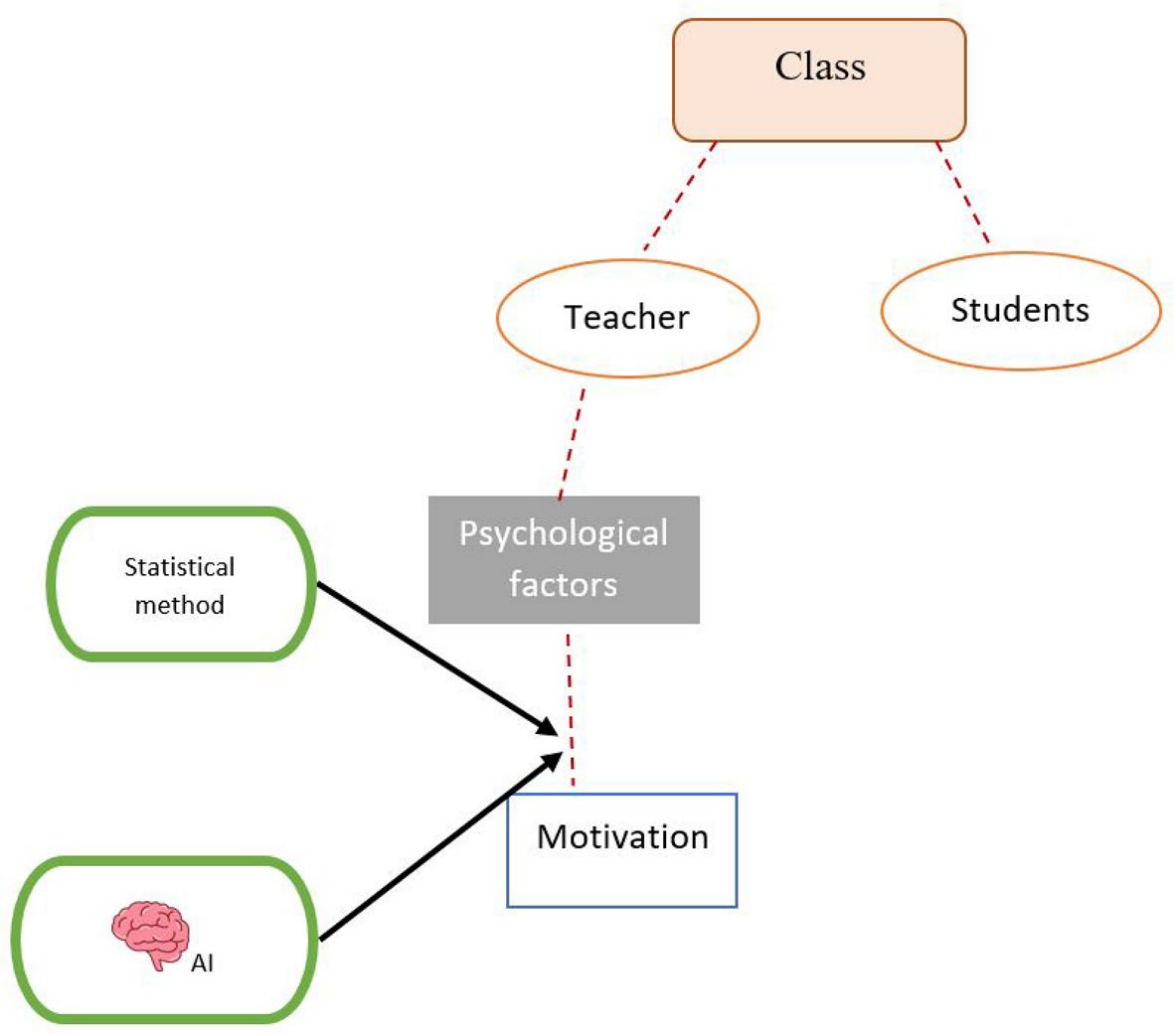
Schematic representation of the study and the integration of the AI and statistical methods.

Figure 1 represents a summary of the study. It indicates that motivation can affect the teachers, and the study investigates the demographic variables of teachers. A very safe way for analyzing the data in this domain and for teaching context is to study them using the statistical methods; however, the researchers of the study also examined the ANFIS method to see how the results differ from the traditional methods and compare the results. Therefore, the current study aims at investigating whether EFL teacher motivation varies by teacher's age, teaching experience, gender, educational level, and academic achievement. Meanwhile, the main concern in this study was to verify the results obtained for a human-related construct with another field of study or, better to say a new technology, which is artificial intelligence (AI), and specifically the ANFIS method.

**Figure 2:**
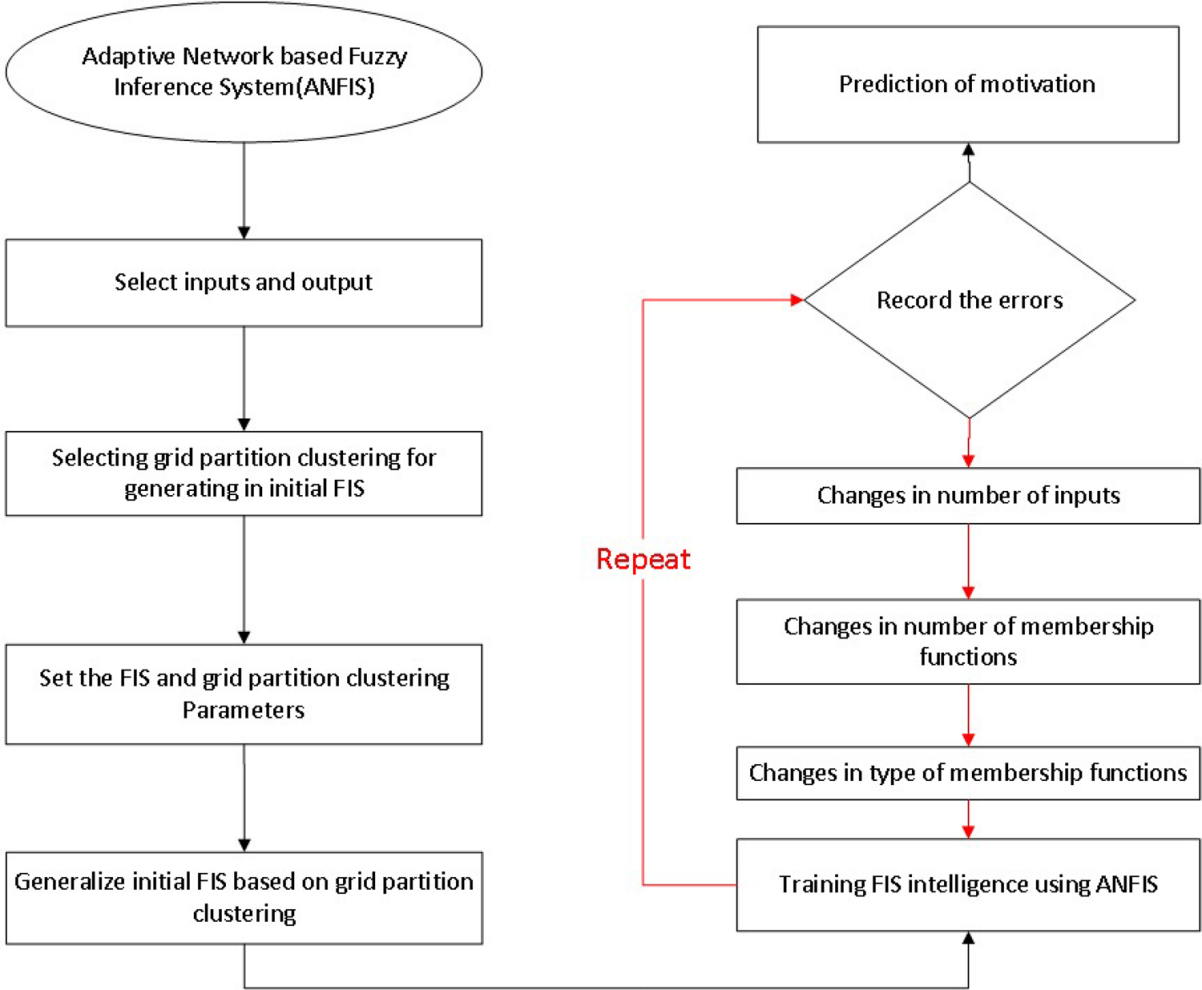
ANFIS flow chart of the AI algorithm in the research, and selection of the model parameter in ANFIS.

The flow chart of the AI algorithm is illustrated in Fig. 2. The figure shows that at the beginning of the method, the input and output parameters are defined in the method as a form of a mathematical matrix. Then in the method, the grid partition clustering is defined for generating the initial Fuzzy interface structure (FIS). In the next stage, the FIS and grid clustering parameters are selected for the method, and the initial FIS is established with proper conditions. After this creation in structure, the training process is started to train the FIS structure and translate all information (inputs and outputs) as a form of FIS structure. To assess the method and the ability to train the error of the system is evaluated in the process. If the error level is high, the code automatically changes the number of input, number/type of membership functions for other training steps. This task is iterated till the deviation reaches an acceptable level. At this stage of the model, the algorithm can predict the motivation.

Artificial intelligence (AI) can react like human beings, including speech recognition, problem-solving, learning, as well as planning. It can also predict human behavior and their psychological condition, such as motivation, by using algorithms. As far as this is a prediction, we need an explanation, but artificial intelligence does not have the ability to explain; therefore, psychology is advantageous. Psychology refers to the study of the mind, and it matters in many professions, such as those dealing with people; therefore, teacher motivation is not an exception. A review of the literature in the realm of teacher motivation indicates a scarcity of research published on teacher motivation, especially its integration with AI. Therefore, the present research explores the relationship of teacher motivation with teacher age, educational level, gender, years of experience, academic achievement, as well as two other aspects of teacher engagement including vigor, and dedication. Indeed, seven research questions are studied, which are as follows:

1. Is there any notable relationship between EFL teacher motivation and age?
2. Is there any notable relationship between EFL teacher motivation and educational level?
3. Is there any notable relationship between EFL teacher motivation and gender?
4. Is there any significant relationship between EFL teacher motivation and years of experience?
5. Is there any significant relationship between EFL teacher motivation and academic achievement?
6. Is there any significant relationship between EFL teacher motivation and vigor?
7. Is there any significant relationship between EFL teacher motivation and dedication?

## Method

### Instrumentation

#### EFL teacher motivation scale (TMS)

For the purpose of measuring teacher motivation, TMS was used, which was designed and validated by Pourtousi and Ghanizadeh^5^. The questionnaire included 42 items, measuring 5 factors. Moreover, Cronbach's alpha was 0.89.

The sample items are “Teachers work together, and they try to build positive environment”, “It is important to be a successful teacher to please school principal or institute supervisor”, and “I am proud of working as a teacher” rated on a five-point Likert scale.

#### Work and Well-Being Survey (UWES)

In order to measure different aspects of engagement, we used the Work and Well-Being Survey (UWES), which was designed by Schaufeli and Bakker^32^. The UWES includes 17 items rated on a 7 point Likert scale. The Cronbach's alpha of UWES is 0.70, and the internal consistency of the scale is suitable.

The sample items are as follows:

“I get carried away when I’m working (absorption)”, “At my job, I am very resilient (vigor)”, and “My job inspires me (dedication)”.

### Participants

The original data relating to participants refer to Pourtousi and Ghanizadeh^5^ paper; however, the analysis that the researchers of the current study decided on a different procedure regarding the data analysis. This study investigates the demographic variables of the EFL teachers and two aspects of their engagement by using ANFIS. Nevertheless, the original study only validated the TMS and found the relationship of motivation with two other psychological factors. The questionnaires were given to 210 English teachers with a variety of age groups from 21 to 42, variety of university of degrees (B.A. holders = 112, M.A. holders = 87, Ph.D. holders = 11). The research had 124 females, 82 males, and the rest did not mention their gender in the questionnaire. The questionnaires were prepared in online and printed formats. The participants were from nine cities of Iran, and mainly from Mashhad, where the main data were gathered. More information regarding the participants can be obtained from the research mentioned above.

In the research procedures, we considered different ethical issues, such as the confidentiality of the data, privacy, and anonymity. The research does not provide any risk to the participants, and the participants were all teachers and colleagues of the researchers who willingly accepted to take part in completing the questionnaires. Therefore, informed consent was obtained from them, and they were provided with information about the study before they participate in the study. Moreover, the whole processes follow the standards of the 1964 Helsinki declaration as well as the ethical standards. Also, the Imam Reza International University research ethics committee approved the study with reference number 599083.

## Findings and discussion

### SPSS results

The descriptive statistics of the teacher motivation scale refers to the information from Pourtousi and Ghanizadeh research study^5^, and the details are as follows:

The mean, standard deviation (Std), minimum and maximum values for teacher motivation (TM) are 143.24, 21.84, 72.00, 212.00, respectively. More information regarding the descriptive statistics can be obtained from the research mentioned above. Moreover, TM has five sub-factors which are Immediate setting (IS), Teacher related (TR), Student related (SR), Administrative related (AR), and Non human-related (NH) factors.

#### Teacher motivation and age

A Pearson Product-Moment correlation was run for examining the correlation between the teacher motivation and age. The results indicate that teacher motivation TM (*r* = − 0.23, *p* < 0.05), and four of the sub factors including NH (*r* = − 0.24, *p* < 0.05), SR (r = − 0.23, *p* < 0.05), IS (*r* = − 0.16, *p* < 0.05), and TR (*r* = − 0.18, *p* < 0.05) correlate with age. However, it was revealed that AR does not correlate with age. The details are shown in Table 1.

**Table 1:** The correlation coefficients of teacher motivation and its five sub-factors with age.

	NH	SR	AR	TR	IS	TM	Age
NH	1						
SR	0.634**	1					
AR	0.613**	0.583**	1				
TR	0.639**	0.615**	0.634**	1			
IS	0.425**	0.524**	0.404**	0.379**	1		
TM	0.835**	0.855**	0.786**	0.857**	0.636**	1	
Age	− 0.240**	− 0.235**	− 0.100	− 0.182**	− 0.163**	− 0.237**	1

**Correlation is significant at the 0.01 level (2-tailed).

#### Teacher motivation and educational level

A one-way *ANOVA* was utilized in order to compare the means of teachers who are grouped based on their university degrees. According to the results, and based on Table 2, teacher motivation(TM) correlates with educational level, and therefore, there are differences among the three groups of teachers regarding their motivation, TM (*F* = 3.63, *p* < 0.05). Also, ANOVA shows that three of the sub-factors have differences in their means, including TR (*F* = 3.92, *p* < 0.05), NH (*F* = 3.79, *p* < 0.05), and SR (*F* = 4.87, *p* < 0.05); however, the exact place of the differences is not obvious. So a post-hoc comparison of the means was run for the three sub-factors in order to find the precise differences. In doing so, Scheffe's test was applied, and Table 3 represents the results of the test. In the following table, and specifically, the column relating to the educational level, the number varies from 1 to 3, that each of them shows a different university degree. Therefore, 1 indicates B.A., 2 indicates M.A., and 3 indicates Ph.D. Also, the results of Scheffe's test do not show any notable differences between the TM mean score of the three degrees. However, the results of the post hoc Scheffe’s test at the level of 0.05 indicated that there is a difference between the first and third groups regarding TR. Also, regarding SR and NH, the first and second groups are different.

**Table 2:** The results of ANOVA for comparison of teacher motivation by university degrees.

	Sum of squares	df	Mean square	F	Sig
**SR**					
Between groups	330.041	2	165.020	4.872	0.009
Within groups	6977.140	206	33.870		
Total	7307.180	208			
**TR**					
Between groups	363.139	2	181.570	3.927	0.021
Within groups	9525.817	206	46.242		
Total	9888.957	208			
**NH**					
Between groups	193.055	2	96.528	3.799	0.024
Within groups	5233.594	206	25.406		
Total	5426.649	208			
AR					
Between groups	27.346	2	13.673	1.054	0.350
Within groups	2671.889	206	12.970		
Total	2699.235	208			
**IS**					
Between groups	16.243	2	8.121	0.615	0.541
Within groups	2718.591	206	13.197		
Total	2734.834	208			
**TM**					
Between groups	2921.642	2	1460.821	3.631	0.028
Within groups	82,872.522	206	402.294		
Total	85,794.164	208

**Table 3:** The Scheffe's test for the comparison of teacher motivation by university degrees.

Dependent variable	(I) educational level	(J) educational level	Mean difference (I–J)	Std. error	Sig.	95% Confidence interval	
						Lower bound	Upper bound
SR	1.00	2.00	2.49571*	0.83600	0.013	0.4344	4.5570
		3.00	2.81302	1.67200	0.245	− 1.3096	6.9356
	2.00	1.00	− 2.49571*	0.83600	0.013	− 4.5570	− 0.4344
		3.00	0.31731	1.65585	0.982	− 3.7654	4.4001
	3.00	1.00	− 2.81302	1.67200	0.245	− 6.9356	1.3096
		2.00	− 0.31731	1.65585	0.982	− 4.4001	3.7654
TR	1.00	2.00	1.46032	0.97683	0.329	− 0.9482	3.8688
		3.00	5.24603*	1.95366	0.029	0.4290	10.0631
	2.00	1.00	− 1.46032	0.97683	0.329	− 3.8688	0.9482
		3.00	3.78571	1.93478	0.150	− .9848	8.5562
	3.00	1.00	− 5.24603*	1.95366	0.029	− 10.0631	− 0.4290
		2.00	− 3.78571	1.93478	0.150	− 8.5562	0.9848
NH	1.00	2.00	1.91670*	0.72405	0.032	0.1315	3.7020
		3.00	2.10439	1.44810	0.350	− 1.4661	5.6749
	2.00	1.00	− 1.91670*	0.72405	0.032	− 3.7020	− 0.1315
		3.00	0.18769	1.43411	0.991	− 3.3483	3.7237
	3.00	1.00	− 2.10439	1.44810	0.350	− 5.6749	1.4661
		2.00	− 0.18769	1.43411	0.991	− 3.7237	3.3483
TM	1.00	2.00	6.35218	2.88120	0.091	− 0.7519	13.4562
		3.00	12.02097	5.76240	0.116	− 2.1871	26.2290
	2.00	1.00	− 6.35218	2.88120	0.091	− 13.4562	0.7519
		3.00	5.66879	5.70672	0.611	− 8.4020	19.7396
	3.00	1.00	− 12.02097	5.76240	0.116	− 26.2290	2.1871
		2.00	− 5.66879	5.70672	0.611	− 19.7396	8.4020

*The mean difference is significant at the 0.05 level.

#### Teacher motivation and gender

An independent samples *t*-test was used for the purpose of examining the significant differences in teacher motivation with different genders. Table 4 shows descriptive statistics of teacher motivation across female as well as male teachers. As shown in the table, the differences are close. Moreover, Table 5 indicates the results of the independent samples *t* test among male and female teachers. As the results indicate, teacher motivation and sub-factors do not differ with gender, and therefore, significant differences are not found between the male and female teachers on their motivation and sub-factors.

**Table 4:** Descriptive statistics of motivation across female and male teachers.

	Gender	N	Mean	Std. deviation	Std. error mean
NH	Female	167	27.9811	5.05826	0.39142
	Male	40	29.1500	5.38540	0.85151
SR	Female	167	39.1986	5.74270	0.44438
	Male	40	39.0333	6.70216	1.05971
AR	Female	167	19.8592	3.38765	0.26214
	Male	40	18.6750	4.42827	0.70017
TR	Female	167	43.6707	6.70681	0.51899
	Male	40	43.4750	7.63926	1.20787
IS	Female	167	18.8503	3.50151	0.27096
	Male	40	18.5976	4.11349	0.65040
TM	Female	167	149.5599	19.60142	1.51680
	Male	40	148.9309	23.31285	3.68608

**Table 5:** Independent samples t-test showing the gender differences for teacher motivation.

	Levene's test for equality of variances		*t*-test for equality of means						
	F	Sig.	t	df	Sig. (2-tailed)	Mean difference	Std. error difference	95% Confidence interval of the difference	
								Lower	Upper
NH	0.148	0.701	− 1.296	205	0.196	− 1.16885	0.90167	− 2.94658	0.60887
SR	0.873	0.351	0.158	205	0.875	0.16527	1.04515	− 1.89535	2.22588
AR	3.434	0.065	1.864	205	0.064	1.18423	0.63527	− 0.06828	2.43673
TR	1.070	0.302	0.161	205	0.872	0.19566	1.21357	− 2.19701	2.58833
IS	0.631	0.428	0.396	205	0.693	0.25266	0.63828	− 1.00578	1.51110
TM	1.899	0.170	0.175	205	0.861	0.62895	3.58400	− 6.43727	7.69518

#### Teacher motivation and teaching experience

A Pearson Product-Moment correlation was utilized for the purpose of examining the relationship between teacher motivation and teaching experience. As shown in Table 6, teacher motivation does not correlate with teaching experience. Two of the sub factors which are NH (*r* = − 0.15*, p* < 0.05), and SR (*r* = − 0.13, *p* < 0.05) correlate with teaching experience, but AR, TR, IS, and TM do not correlate with teaching experience.

**Table 6:** The correlation coefficients of teacher motivation and its five sub-factors with teaching experience.

	NH	SR	AR	TR	IS	TM	Experience
NH	1						
SR	0.634**	1					
AR	0.613**	0.583**	1				
TR	0.639**	0.615**	0.634**	1			
IS	0.425**	0.524**	0.404**	0.379**	1		
TM	0.835**	0.855**	0.786**	0.857**	0.636**	1	
Experience	− 0.155**	− 0.137**	− 0.034	− 0.078	− 0.055	− 0.121	1

**Correlation is significant at the 0.01 level (2-tailed).

#### Teacher motivation and academic achievement

A Pearson Product-Moment correlation was utilized in the study in order to study any significant correlations between teacher motivation and GPA. According to Table 7, teacher motivation does not correlate with academic achievement (*p* < 0.05). But two of the sub-factors correlate with it, which are AR (*r* = 0.22, *p* < 0.05) and TR (*r* = 0.15*, p* < 0.05) correlate with teacher academic achievement.

**Table 7:** The correlation coefficients of teacher motivation and its five sub-factors with academic achievement.

	NH	SR	AR	TR	IS	TM	Academic achievement
NH	1						
SR	0.634**	1					
AR	0.613**	0.583**	1				
TR	0.639**	0.615**	0.634**	1			
IS	0.425**	0.524**	0.404**	0.379**	1		
TM	0.835**	0.855**	0.786**	0.857**	0.636**	1	
Academic achievement	0.085	− 0.010	0.223**	0.156**	0.049	0.119	1

**Correlation is significant at the 0.01 level (2-tailed).

#### Teacher motivation and vigor

A Pearson Product-Moment correlation was utilized in the study in order to study any significant correlation between teacher motivation and vigor. As indicated in Table 8, teacher motivation and its sub factors correlate with vigor. So TM (*r* = 0.28, *p* < 0.05), IS (*r* = 0.22, *p* < 0.05), and TR (r = 0.35, *p* < 0.05), AR (r = 0.21, *p* < 0.05), SR (*r* = 0.14, *p* < 0.05), and NH (*r* = 0.19, *p* < 0.05) correlate with vigor.

**Table 8:** The correlation coefficients of teacher motivation and its five sub-factors with vigor.

	NH	SR	AR	TR	IS	TM	Vigor
NH	1						
SR	0.634**	1					
AR	0.613**	0.583**	1				
TR	0.639**	0.615**	0.634**	1			
IS	0.425**	0.524**	0.404**	0.379**	1		
TM	0.835**	0.855**	0.786**	0.857**	0.636**	1	
Vigor	0.191**	0.147**	0.212**	0.356**	0.225**	0.288**	1

**Correlation is significant at the 0.01 level (2-tailed).

#### Teacher motivation and dedication

A Pearson Product-Moment correlation was utilized in the study in order to study any significant correlation between teacher motivation and dedication, and the details are shown in Table 9. Based on the results, TM (*r* = 0.2, *p* < 0.05), IS (*r* = 0.17, *p* < 0.05), TR (r = 0.31, *p* < 0.05), and AR (r = 0.15, *p* < 0.05) correlate with dedication. However, SR, and NH do not correlate with dedication.

**Table 9:** The correlation coefficients of teacher motivation and its five sub-factors with dedication.

	NH	SR	AR	TR	IS	TM	Dedication
NH	1						
SR	0.634**	1					
AR	0.613**	0.583**	1				
TR	0.639**	0.615**	0.634**	1			
IS	0.425**	0.524**	0.404**	0.379**	1		
TM	0.835**	0.855**	0.786**	0.857**	0.636**	1	
Dedication	0.103	0.062	0.159**	0.310**	0.176*	0.208**	1

**Correlation is significant at the 0.01 level (2-tailed).

Table 10 shows a sample of the data of the study and how the data were used in the ANFIS. Generally, there were five inputs in the study. The first three inputs were age, academic achievement, and years of experience. The next two inputs were educational level and gender, but the intelligence was not enough, the last two inputs were substituted with two other inputs, which were vigor and dedication to optimize the system, and the output was motivation.

**Table 10:** Sample data of the inputs and output of the study.

Number of sample data	Input 1	Input 2	Input 3	Input 4	Input 5	Output
1	22	19	2	25	18	159
2	33	19	6	31	21	148
3	23	16	4	20	17	144
4	23	16	9	24	19	146
5	28	15	9	11	8	145
6	23	16	2	20	13	141
7	25	15	3	23	13	133
8	33	15	5	26	18	133
9	32	17	6	22	14	132
10	21	19	1	30	24	152
11	25	15	3	23	13	133
12	33	15	5	26	18	133

### ANFIS results

**Figure 3:**
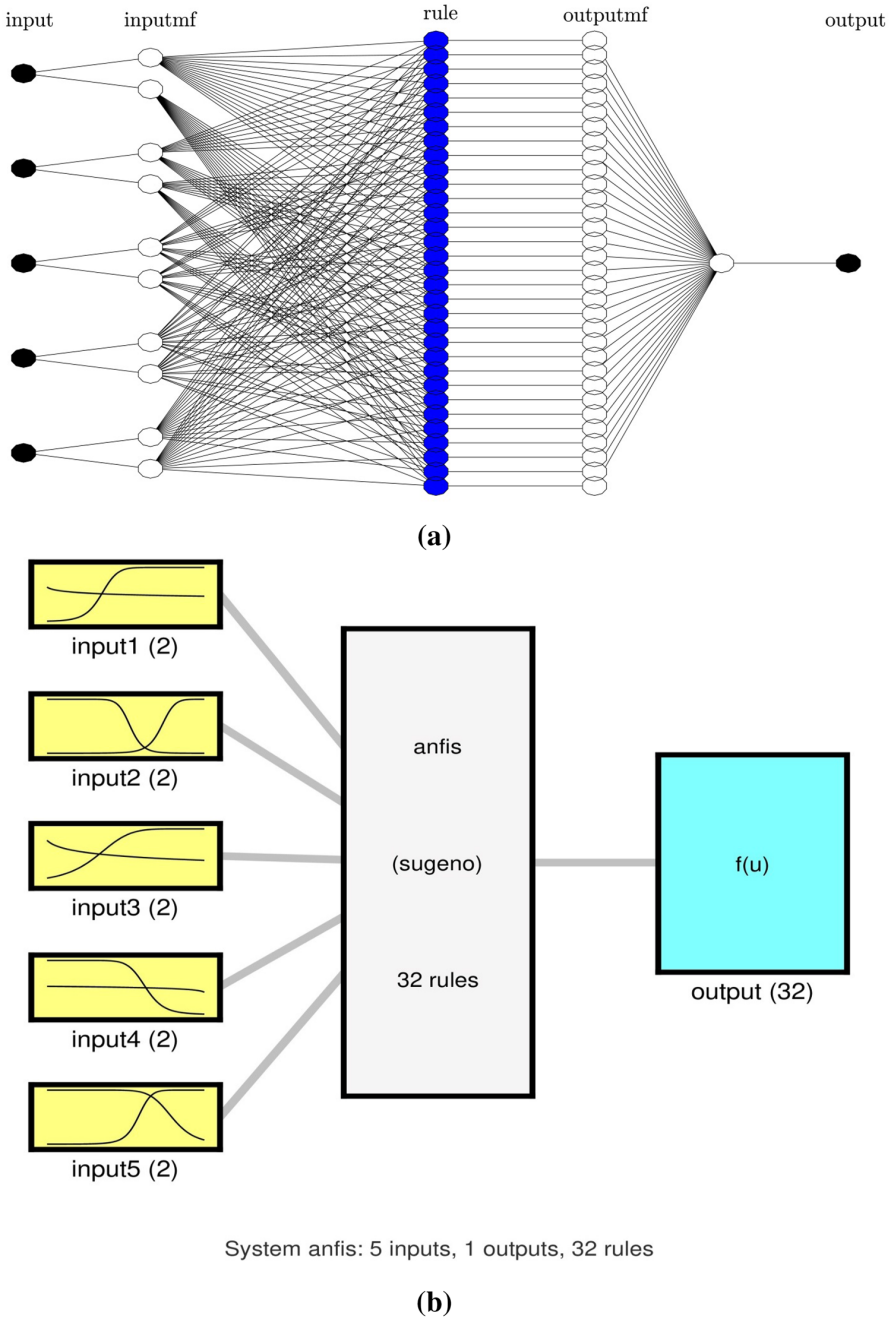
(**a**) ANFIS structure and pattern of input parameters (connection between input parameters) for five inputs and number of MFs = 2. (**b**) Structure and distribution of membership functions in each input parameter.

In the current study, five parameters were used as the input of the ANFIS, including age, GPA, teaching experience, vigor, and dedication, respectively, and the study included one output which is teacher motivation (see Fig. 3a,b).

**Figure 4:**
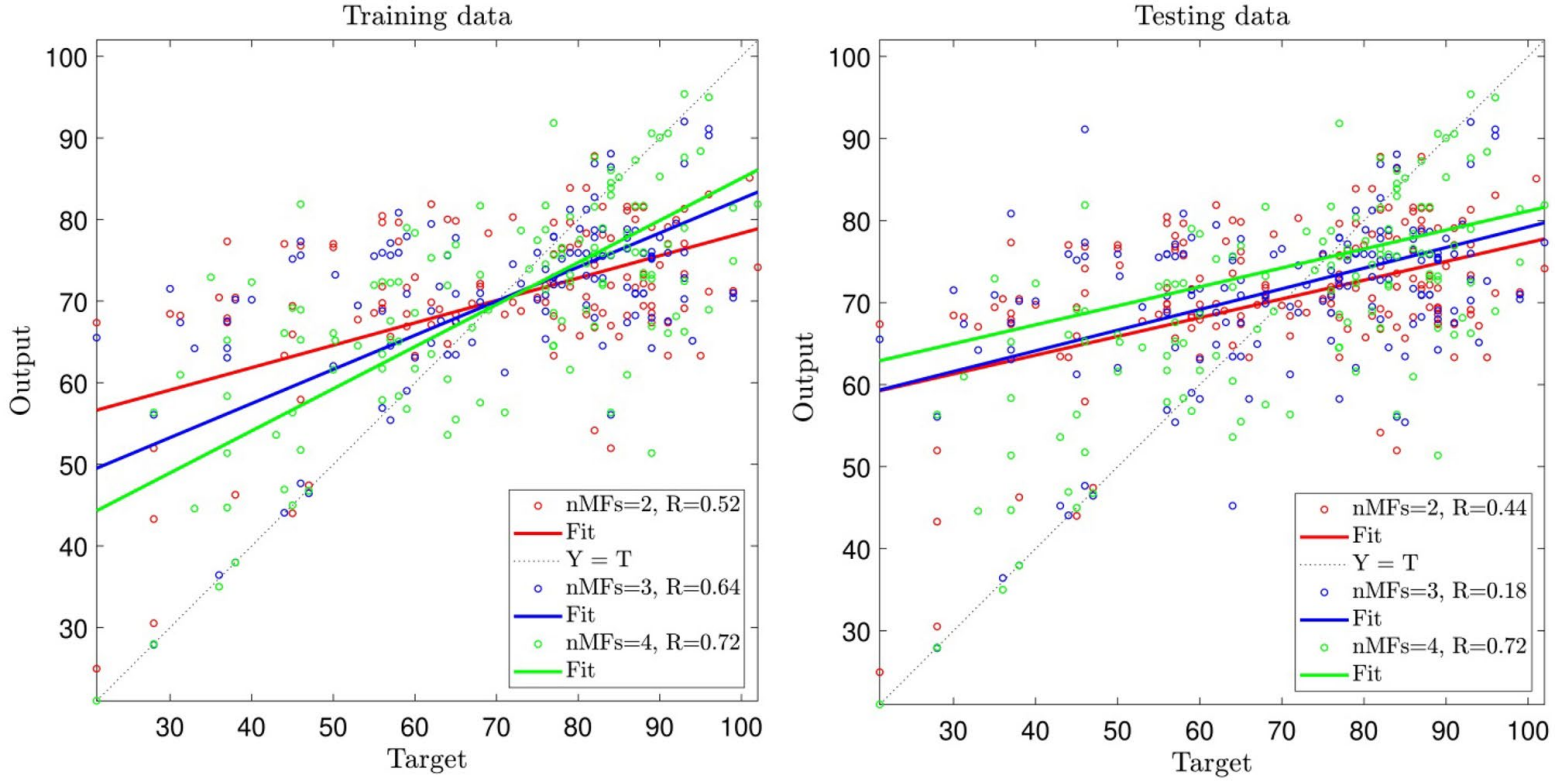
Ability of training and testing processes for number of MFs = 2, 3, 4; two inputs; *gbellmf* (membership function).

In this study, the maximum iteration was equal to 500, and P representing the number of data that were engaged in the training process was equal to 70%, and the kind of membership functions (MFs) is *gbellmf* standing for generalized bell-shaped membership function. To scrutinize the ANFIS method at the beginning of the learning process, which includes training and testing, two inputs were analyzed. When the number of MFs = 2, the R-value for the training and testing evaluation steps is 0.52, 0.44, respectively (see Fig. 4).

For increasing the accuracy and capability of the ANFIS model, the number of MFs was analyzed. According to Fig. 4, when the number of membership functions in each input parameter is equal to 3, R-value is 0.64 for the training, and this value for the testing process reduces to 0.018. Increment of membership function in inputs from three to four was also analyzed, and R-value reaches 0.72 for the training and testing processes. The results also showed that the level of the model’s accuracy and prediction capability was not enough to predict motivation. In this regard, the number of input parameters should be increased to improve the level of accuracy.

**Figure 5:**
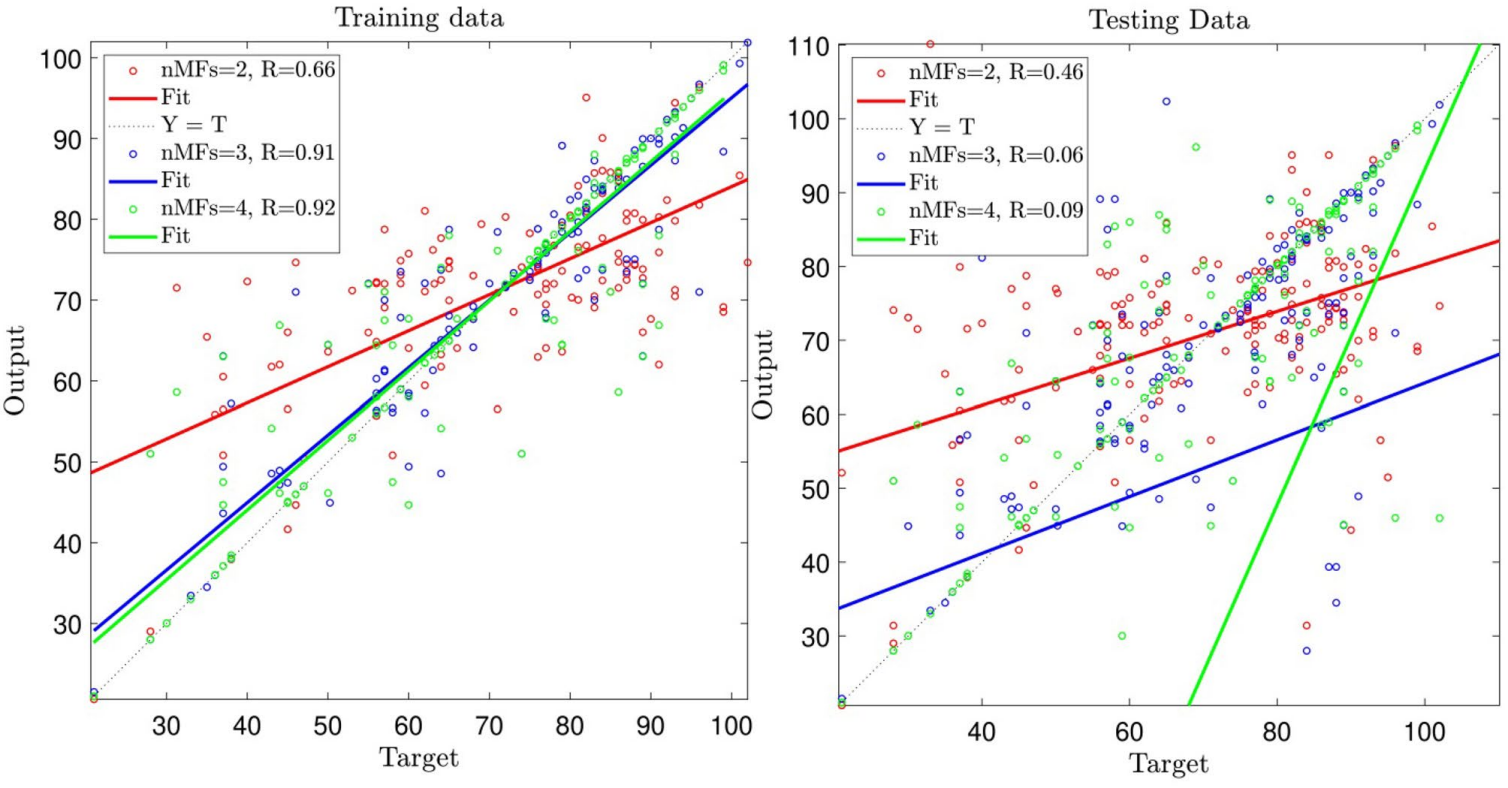
Ability of training and testing processes for number of MFs = 2, 3, 4; three inputs; *gbellmf* (membership function).

This level of accuracy and prediction capability is not enough for the ANFIS model, so the number of MFs increased to 4. According to Fig. 5, when MFs equals 3, the R-value relating to the training process equals 0.91, and the R-value relating to the testing process equals 0.06. Moreover, when the number MFs = 4, R-value is equal to 0.92 for the training process and R = 0.09 for the testing process showing that increasing the number of MFs does not significantly influence the increase of intelligence of the system. In this case, still increasing the number of inputs was required for enhancing the level of accuracy.

**Figure 6:**
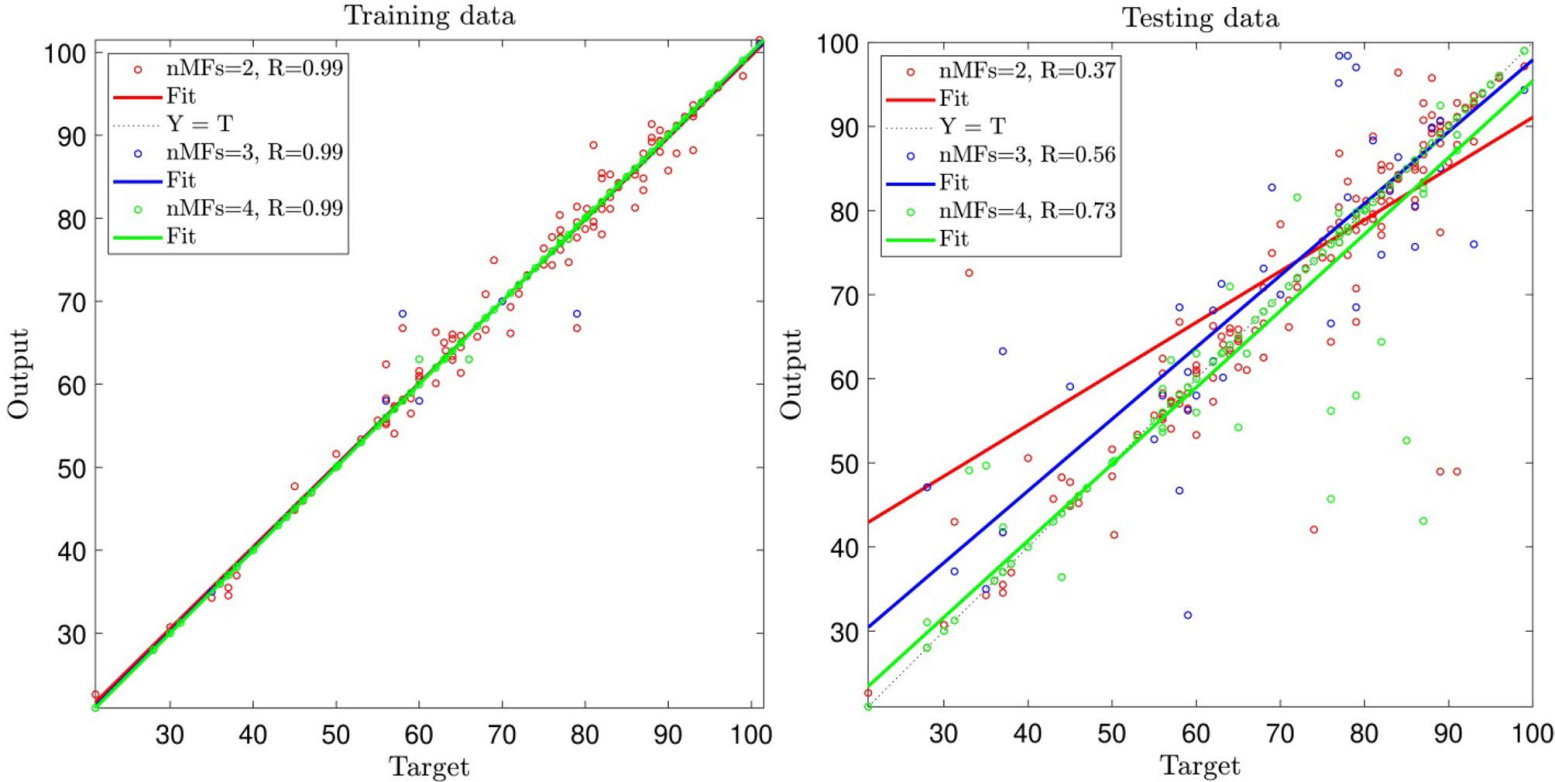
Ability of training and testing processes for MFs = 2, 3, 4; four inputs; *gbellmf* (membership function).

After that, the number of inputs increased from 3 to 4, and MFs to 2, 3, and 4, and the processes of learning include training and testing. Figure 6 shows the accuracy and prediction capability of the model for processes of training and testing. The figure shows that by increasing the number of inputs to 4, the R-value for the training process increases and reaches 0.99. However, R-value for the testing just enhances and reaches an acceptable value. This means that the number of inputs must be increased to see the changes in the system.

**Figure 7:**
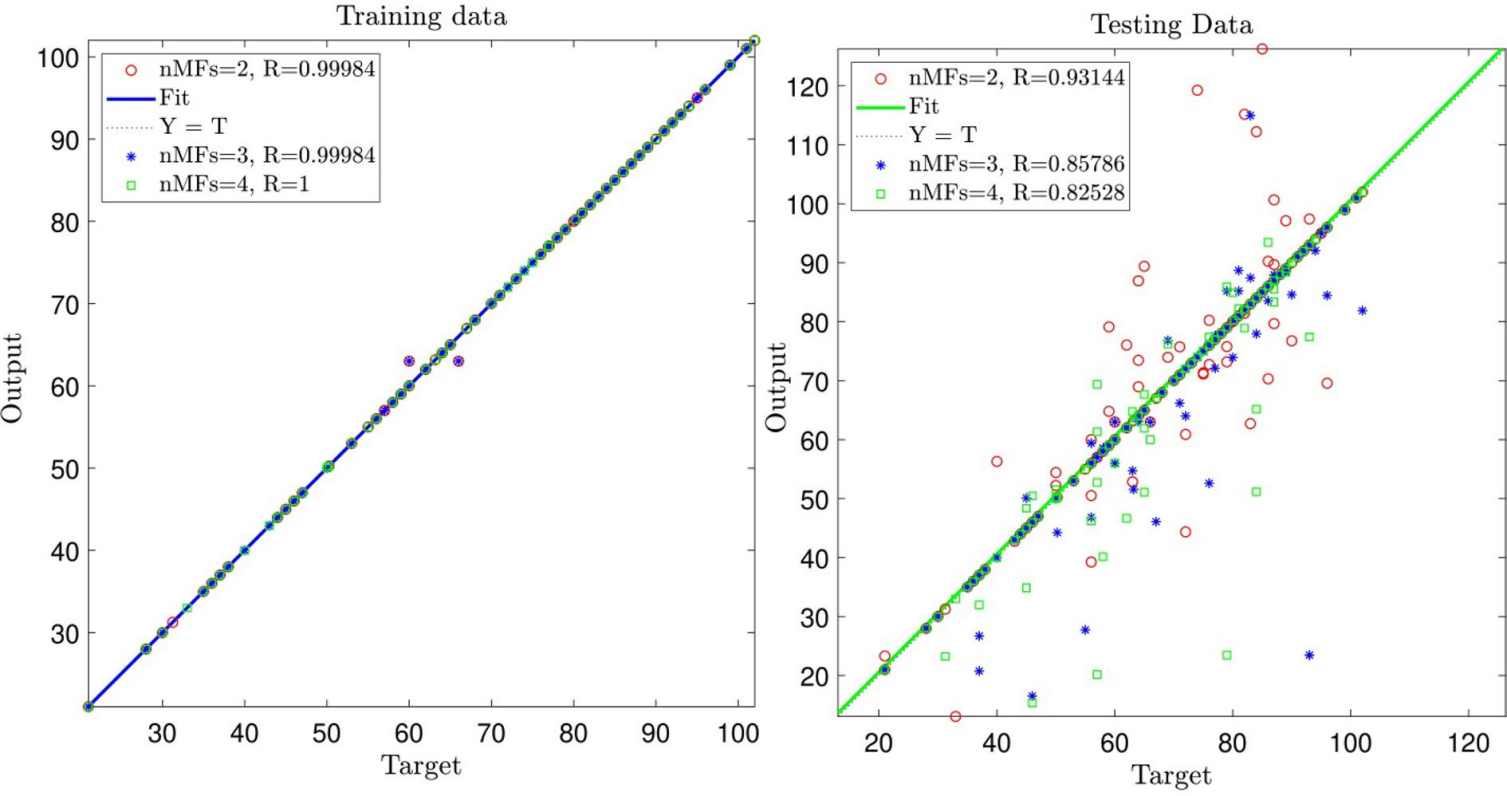
Ability of training and testing processes for number of MFs = 2, 3, and 4; five inputs; *gbellmf* (membership function).

The intelligence or capability of prediction significantly increased by increasing the input in the models, particularly when the number of inputs rises from four to five and applying a different number of membership functions at each input parameter. Additionally, the impact of different membership functions is examined to find out the best level of accuracy for the AI model. The results show that by an increment of membership functions in the input parameter, the accuracy of the model reduces. Therefore, for the current research, the number of membership functions two can be a good candidate to train datasets, particularly with five input parameters (see Fig. 7). This finding can illustrate the importance of the tuning model parameters for the ANFIS method to build up the AI model with a high level of repeatability. In addition, as a recommendation using a high number of membership functions cannot build an accurate model with high prediction capability. In addition to that, adding more functions in each input can make the model more expensive.

**Figure 8:**
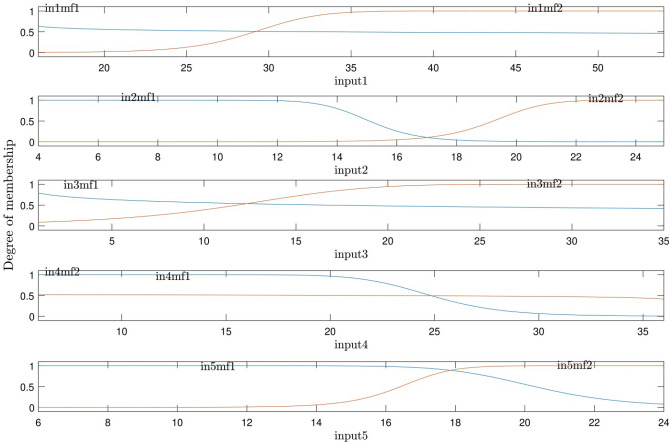
Degree of inputs membership functions for five input parameters.

Different degrees of membership functions are shown in Fig. 8. Generally, the model is scalable for the particular range of input parameters, as shown in this figure. To predict the process for the wider range of input parameters, more inputs are required in the training process.

**Figure 9:**
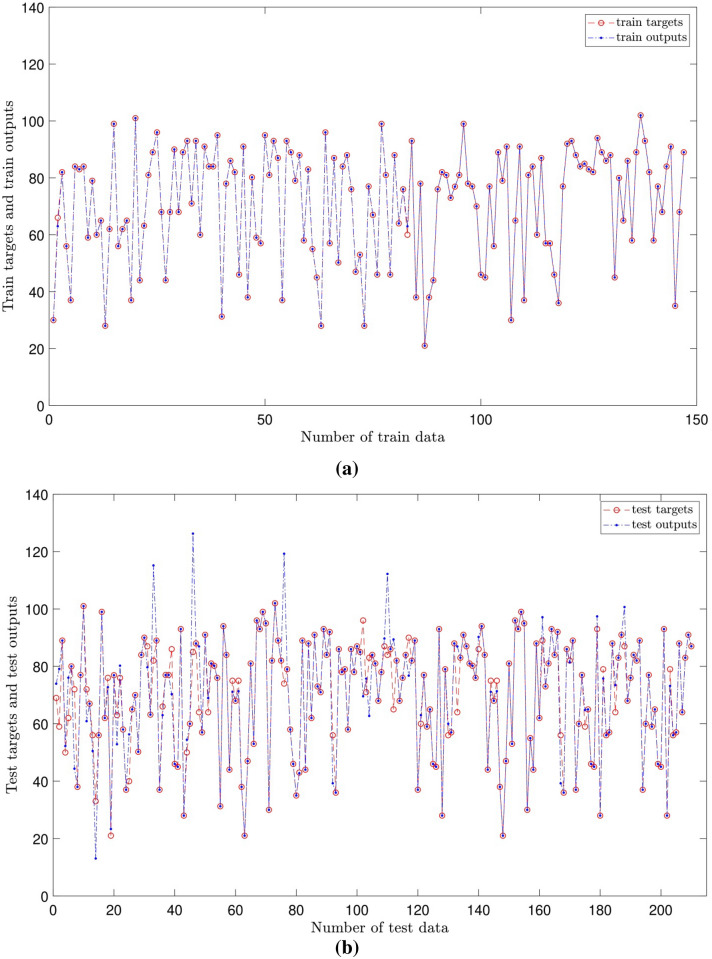
(**a**) Comparison between prediction results and actual values (training evaluation process); [number of MFs = 2; five inputs; *gbellmf* (membership function)]. (**b**) Comparison between prediction results and actual values (testing evaluation process); [number of MFs = 2; five inputs; gbellmf (membership function)].

In Fig. 9a,b, prediction (ANFIS results) and actual values for two different evaluation steps, including training and testing, are shown. The two figures compare the difference between the training testing processes when the output of the study is motivation, and five inputs are added to the system. The results show that the ANFIS model can perfectly track target values in the domain for the training and testing process.

**Figure 10:**
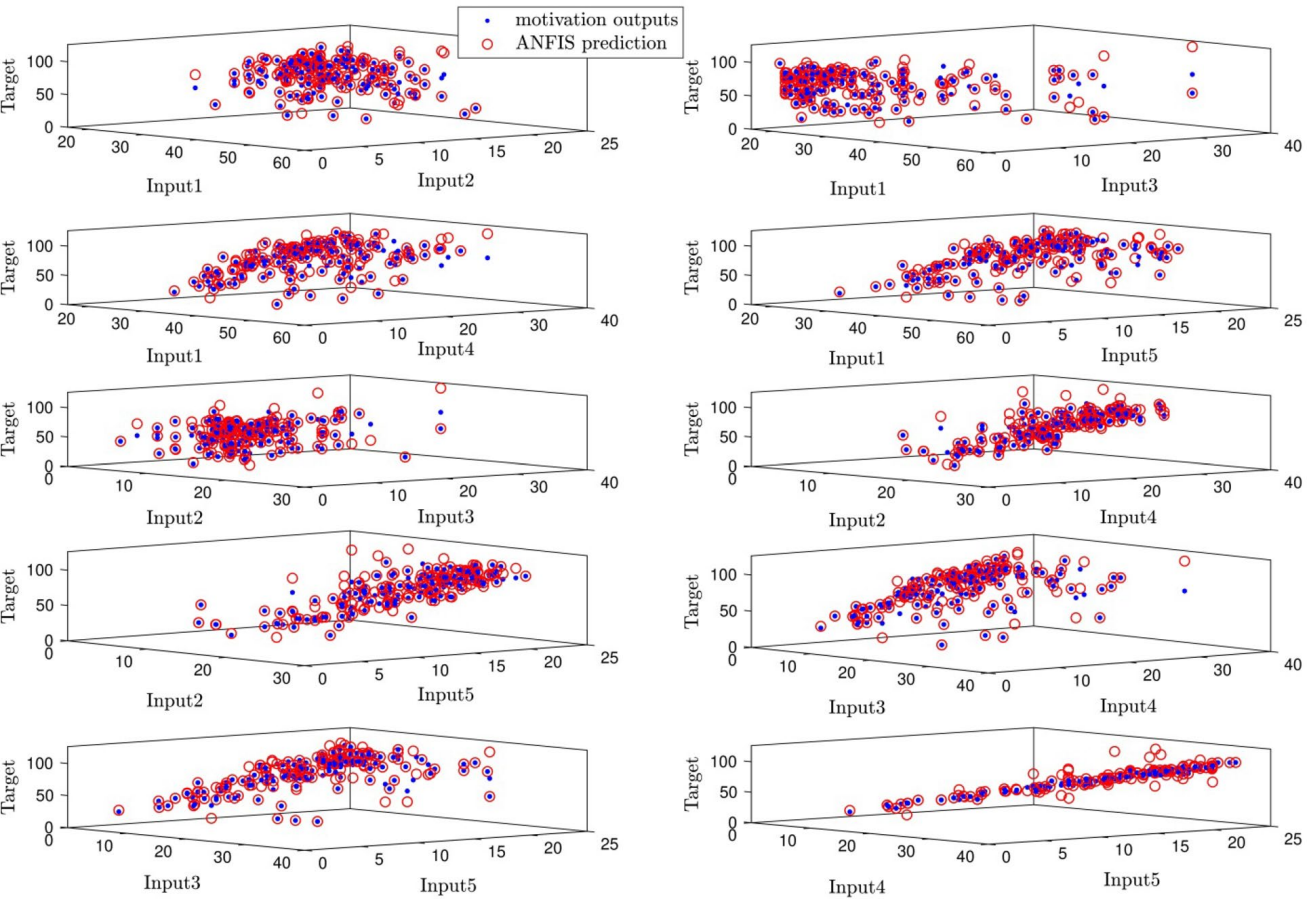
ANFIS targets and outputs data correlation.

According to Fig. 10, ANFIS target and ANFIS output are in good agreement with each other by considering different inputs. This means that ANFIS has a good prediction for the current dataset, and its algorithms can predict the output.

**Figure 11:**
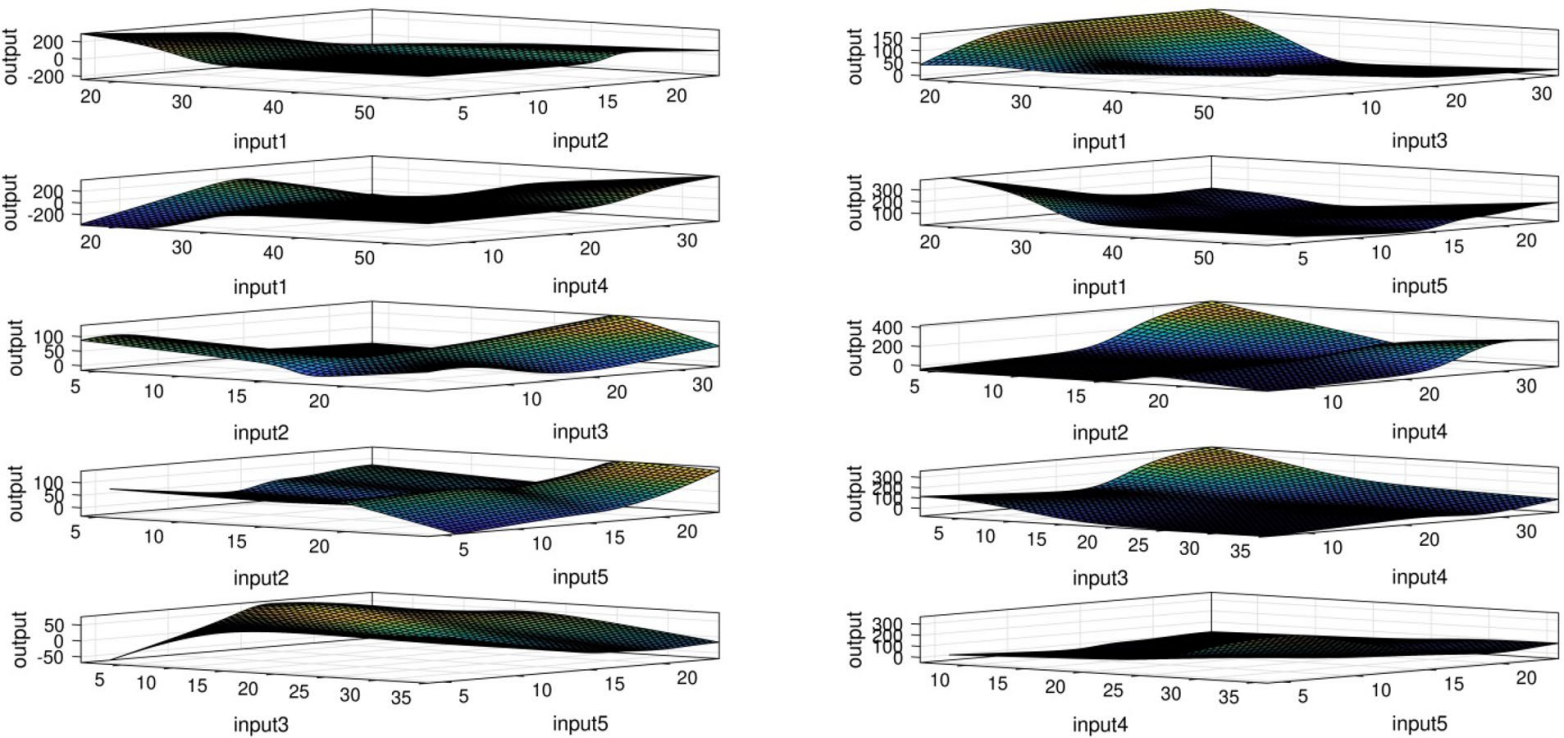
ANFIS prediction surface.

Artificial intelligence has the capacity to use many nodes in order to predict what has not been existed in the learning process that is presented in Fig. 11. This means that by deleting some study features or some of the inputs from the training stage of ANFIS, the system still has the prediction capability.

**Figure 12:**
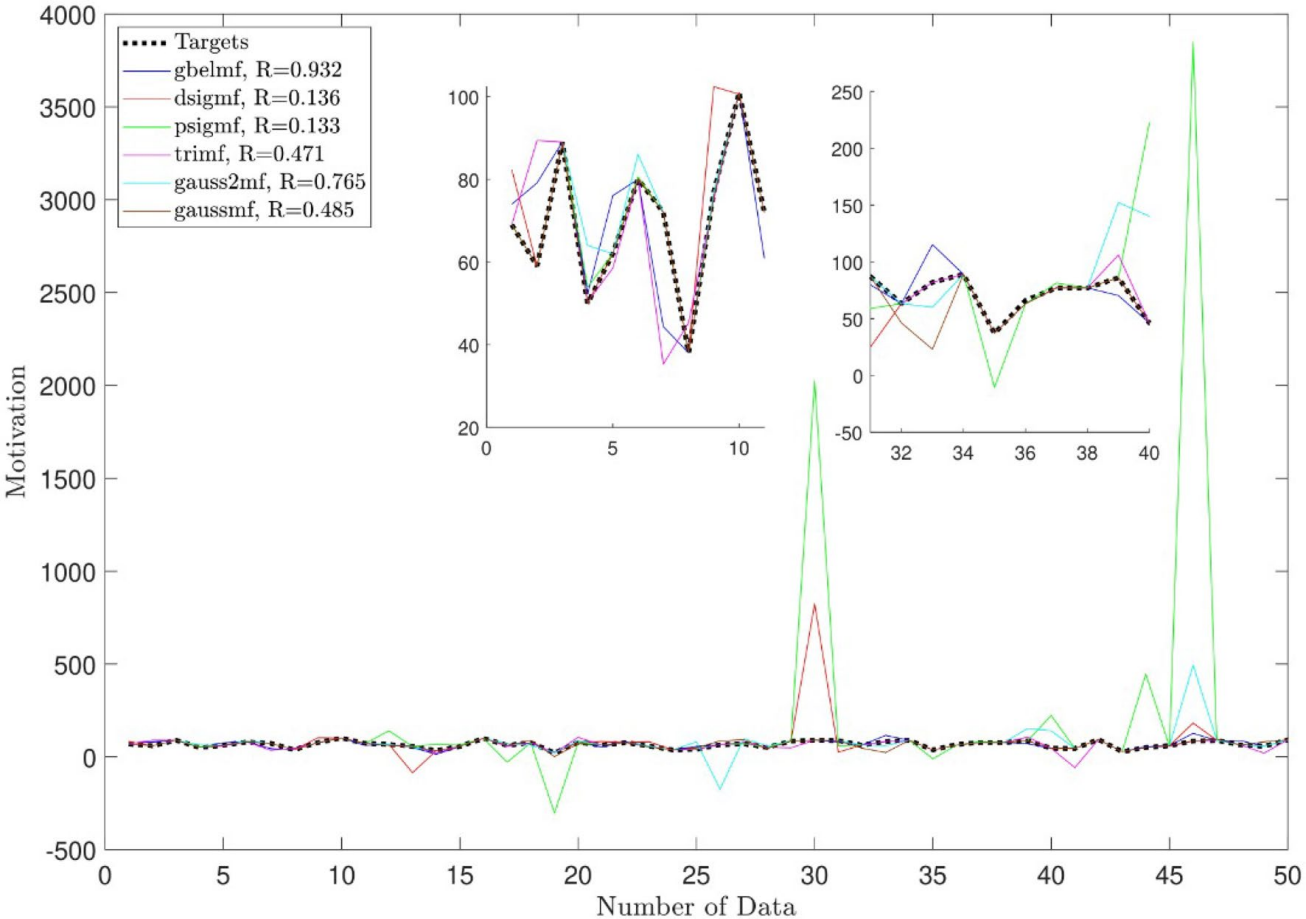
Impact of different membership functions on the accuracy of the model and prediction capability.

For further evaluation of the current AI methodology, different membership functions are also selected for the prediction. In this regard, all membership functions are used and compared with the current methodology. Figure 12 shows that *psig* and *dsig* membership functions contain a lower level of the model’s accuracy and prediction capability. However, the *gbell* function shows a good level of accuracy (R > 0.93). This method can also predict and track the motivation level in a similar range of reality than other membership functions in the ANFIS method.

**Figure 13:**
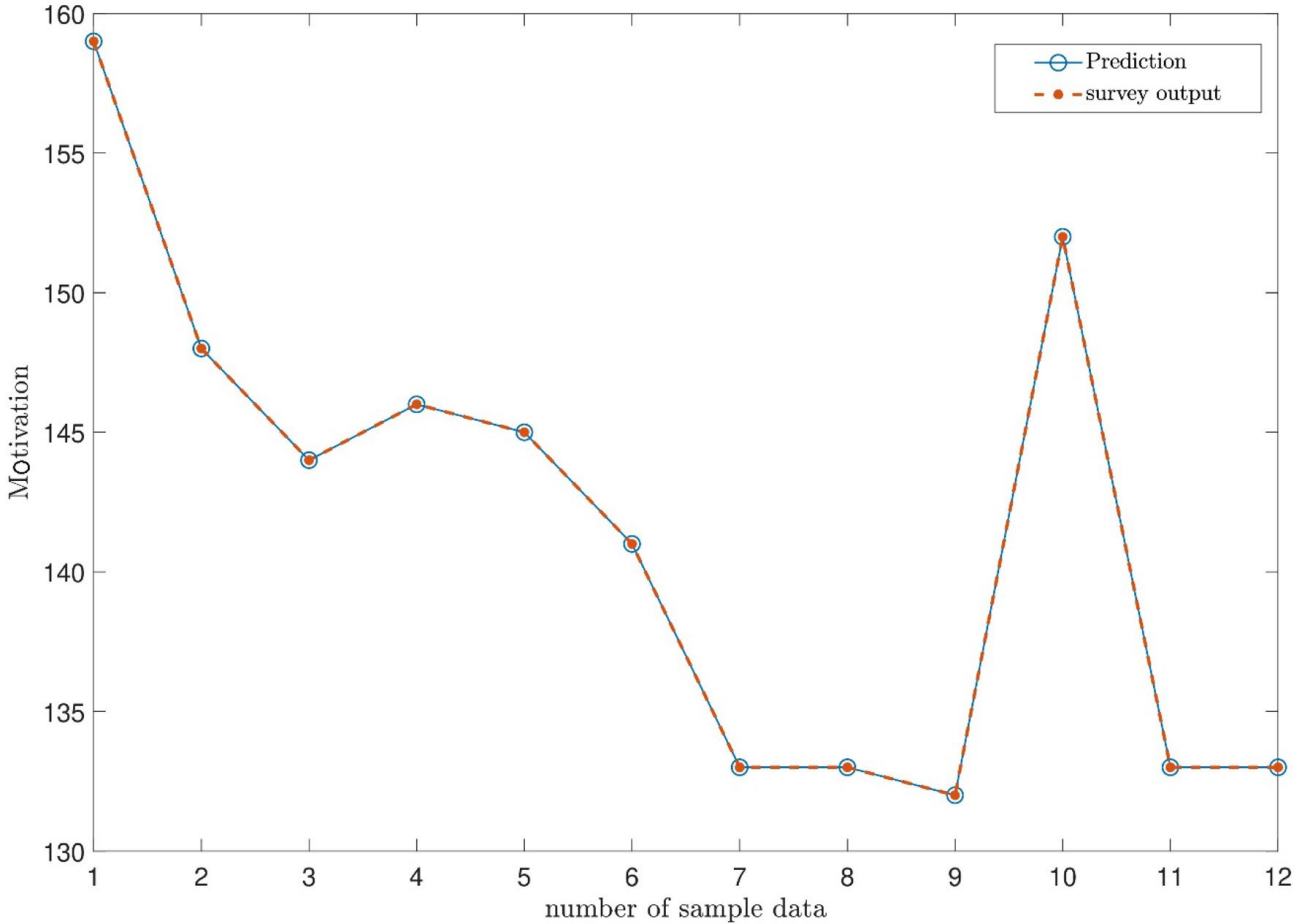
Prediction of the sample data for teacher motivation.

Figure 13 shows the prediction of the output based on Table 10. So from the whole data, 12 inputs of 12 participants were inserted into the system to see whether ANFIS can predict the data. As the figure shows, ANFIS could accurately predict the output that is motivation, and it is based on the inputs engaged in the study.

The present research paper examined the relationship between teacher motivation and demographic variables, including age, educational level, gender, experience, academic achievement, and two other aspects, including vigor and dedication. Regarding the first question examining whether teacher motivation varies by the teacher's age, the findings showed a significant link between the variables. Teacher age was a significant predictor of teacher motivation in another study. Martin and Shoho stated that the teacher's attitudes and beliefs change and are controlled by the teacher's age^33^. The next question examined whether teacher motivation varies by the university degree of teachers, no significant difference was found among different groups of the teachers regarding the total teacher motivation. But for some of the sub factors, the results were different. Regarding the third research question, which investigated whether teacher motivation varies by having a different gender, no significant differences was found among the participants. Another study stated that female teachers experience higher levels of classroom and workload stress, which is contrary to our results as gender was not one of the factors that determines teacher motivation^34^. The fourth research question investigated whether teacher motivation varies with their teaching experience, and the results revealed that teacher motivation did not correlate with teaching experience except for two of the sub-factors. According to the results, teacher motivation cannot be determined by experience. The results of the study are in line with the findings of other studies. For instance, a research study indicated that a typical or average teacher with higher experience is necessarily not more effective than a teacher with fewer years of experience^35^. Also, Martin and Shoho mentioned that the boundary between novice and experienced teachers is not exactly defined^33^. Moreover, the fifth research question investigated whether teacher motivation varies by their own academic achievement. The results indicated that teacher motivation does not correlate with academic achievement. In other words, teachers' academic achievement or their GPA do not affect their motivation. The final aspects of teachers investigated in the study were vigor and dedication, which are parts of engagement. The study revealed a positive correlation between motivation and these two aspects meaning that higher engagement, specifically vigor and dedication parts, can lead to higher motivation. These two aspects played a significant role in the ANFIS model for better prediction. In this regard, Skaalvik and Skaalvik^36^ mentioned that teacher well-being could have a predicting role in a higher level of engagement as well as a lower of level motivation when teachers are willing to leave teaching, and therefore, the current study found the relationship of motivation as well as engagement. Furthermore, the ANFIS model indicated that by adding five inputs to the system, which were all of the variables in the study, the system could have high accuracy and prediction capability.

## Conclusions

The problem with machine learning and artificial intelligence is that it has an association function problem. Due to the dependence of the technology on large-scale data and numerical values, in some aspects, it lacks association function, which is similar to the human brain^21^. The results which are driven from ANFIS indicated when all five inputs are engaged in the process of learning, the intelligence increases as well. This is true in real life because psychological aspects are decided by the whole variables and not one of them. In SPSS, which is mostly used in psychology fields, each factor is analyzed one by one, but ANFIS added each of the factors incrementally, and therefore we could see when it becomes intelligent. After adding all of the inputs, the accuracy and prediction capability of the ANFIS model increased significantly. This is because the neural networks increased in the system, and the statistical approaches do not have the capability to have more and more neural networks in each phase. However, it is evident that we need large-scale data to have better, comprehensive results, and this study only used a sample. In addition, in the five input parameters, a small number of membership functions (such as two) at each input can make the model more accurate than larger numbers (such as four). This finding can also highlight the importance of sensitivity analysis on the AI model’s parameter before creating a prediction toolbox.

Using the intelligence obtained from the ANFIS method can help us to predict those nodes that are engaged in motivation or better in the psychological aspects of humans. Therefore, we can predict some hidden aspects of teacher motivation by using AI. Based on the algorithms, we can predict the different aspects. Moreover, the current study shows another data analysis method, which is different from the traditional analysis in this field.

ANFIS model showed that by adding each input to the system, the accuracy of the system increased that could be like a human. By increasing the neural networks, the system reached an acceptable level of accuracy. The findings indicated other research studies could be developed in social sciences, humanities, and psychology mixed with soft computing methods and AI algorithms. Therefore, this can open new doors to the researchers.

Generally, when a new method is used in a study, some new insights can be achieved, and the new method can have various advantages. Therefore, the computational time for running AI models could be decreased to the minimum. Furthermore, some AI models and their algorithms are based on natural phenomena^37^, and if some new concepts to be tested in them, AI could have high prediction capability and accuracy. Furthermore, in AI, big data can be analyzed^21^. It is worth mentioning that each method has its own benefits. They can be used side by side to compare data and complete each other.

## References

[1] Gardner R (1985). Social Psychology and Second Language Learning: The Role of Attitudes and Motivation.

[2] Pourtoussi Z, Ghanizadeh A, Mousavi V (2018). A Qualitative in-depth analysis of the determinants and outcomes of EFL teachers’ motivation and demotivation. Int. J. Instr..

[3] Gardner RC, Lalonde RN, Moorcroft R (1985). The role of attitudes and motivation in second language learning: Correlational and experimental considerations. Lang. Learn..

[4] Dornyei, Z. The psychology of the language learner: Individual differences in second language acquisition. *New Jersey Mahwah* (2005).

[5] Pourtousi Z, Ghanizadeh A (2020). Teachers’ motivation and its association with job commitment and work engagement. Psychol. Stud. (Mysore).

[6] Havik T, Westergård E (2020). Do teachers matter? Students’ perceptions of classroom interactions and student engagement. Scand. J. Educ. Res..

[7] Thoonen EEJ, Sleegers PJC, Oort FJ, Peetsma TTD, Geijsel FP (2011). How to improve teaching practices. Educ. Admin. Q..

[8] Arifin HM (2015). The influence of competence, motivation, and organisational culture to high school teacher job satisfaction and performance. Int. Educ. Stud..

[9] Steren B, Antunes DD, José J, Mosquera M, Stobäus CD (2016). Teachers’ motivation related to teaching and learning processes. Creat. Educ..

[10] Kocabaş İ (2009). The effects of sources of motivation on teachers’ motivation levels. Education.

[11] Alam MT, Farid S (2011). Factors affecting teachers’ motivation. Int. J. Bus. Soc. Sci..

[12] Pelletier LG, Séguin-Lévesque C, Legault L (2002). Pressure from above and pressure from below as determinants of teachers’ motivation and teaching behaviors. J. Educ. Psychol..

[13] Heinz M (2015). Why choose teaching? An international review of empirical studies exploring student teachers’ career motivations and levels of commitment to teaching. Educ. Res. Eval..

[14] Khalil R (2020). The sudden transition to synchronized online learning during the COVID-19 pandemic in Saudi Arabia: A qualitative study exploring medical students’ perspectives. BMC Med. Educ..

[15] Subedi S, Nayaju S, Subedi S, Shah SK, Shah JM (2020). Impact of E-learning during COVID-19 pandemic among nursing students and teachers of Nepal. Int. J. Sci. Healthc. Res..

[16] Mahmood S (2020). Instructional strategies for online teaching in COVID-19 pandemic. Hum. Behav. Emerg. Technol..

[17] Baber H (2020). Determinants of students’ perceived learning outcome and satisfaction in online learning during the pandemic of COVID19. J. Educ. e-Learn. Res..

[18] Adnan M, Anwar K (2020). Online learning amid the COVID-19 pandemic: Students’ perspectives. J. Pedagog. Sociol. Psychol..

[19] Alawamleh M, Al-Twait LM, Al-Saht GR (2020). The effect of online learning on communication between instructors and students during Covid-19 pandemic. Asian Educ. Dev. Stud..

[20] Mishra L, Gupta T, Shree A (2020). Online teaching-learning in higher education during lockdown period of COVID-19 pandemic. Int. J. Educ. Res. Open.

[21] Lu H, Li Y, Chen M, Kim H, Serikawa S (2018). Brain intelligence: Go beyond artificial intelligence. Mob. Netw. Appl..

[22] Kumar V, Dixit A, Javalgi RG, Dass M (2016). Research framework, strategies, and applications of intelligent agent technologies (IATs) in marketing. J. Acad. Mark. Sci..

[23] Brill TM, Munoz L, Miller RJ (2019). Siri, Alexa, and other digital assistants: A study of customer satisfaction with artificial intelligence applications. J. Mark. Manag..

[24] Sweetser P, Wiles J (2002). Current AI in games: A review. Aust. J. Intell. Inf. Process. Syst..

[25] Ali W (2017). Phishing website detection based on supervised machine learning with wrapper features selection. Int. J. Adv. Comput. Sci. Appl..

[26] Jiang F (2017). Artificial intelligence in healthcare: Past, present and future. Stroke Vasc. Neurol..

[27] Luxton DD (2014). Artificial intelligence in psychological practice: Current and future applications and implications. Prof. Psychol. Res. Pract..

[28] Takagi T, Sugeno M (1985). Fuzzy identification of systems and its applications to modeling and control. IEEE Trans. Syst. Man. Cybern..

[29] Moghadasin M (2020). Psychology with soft computing method: Forecasting of anger expression of the human using the developed model based on support vector machine. TPM Test. Psychom. Methodol. Appl. Psychol..

[30] Çakıt E, Karwowski W, Murata A, Olak AJ (2020). Application of structural equation modeling (SEM) and an adaptive neuro-fuzzy inference system (ANFIS) for assessment of safety culture: An integrated modeling approach. Safety.

[31] Çakıt E (2020). Assessing safety at work using an adaptive neuro-fuzzy inference system (ANFIS) approach aided by partial least squares structural equation modeling (PLS-SEM). Int. J. Ind. Ergon..

[32] Schaufeli, W. & Bakker, A. *UWES: Utrecht Work Engagement Scale: Preliminary Manual* (Utr. Occup. Heal. Psychol. Unit, Utr. Univ., 2004).

[33] Martin, N. K. & Shoho, A. R. Teacher Experience, Training, and Age: The Influence of Teacher Characteristics on Classroom Management Style Creating (2000).

[34] Klassen RM, Chiu MM (2010). effects on teachers’ self-efficacy and job satisfaction: Teacher gender, years of experience, and job stress. J. Educ. Psychol..

[35] Ladd HF, Sorensen LC (2017). Returns to teacher experience: Student achievement and motivation in middle school. Educ. Financ. Policy.

[36] Skaalvik EM, Skaalvik S (2018). Job demands and job resources as predictors of teacher motivation and well-being. Soc. Psychol. Educ..

[37] Chazelle, B. Natural algorithms. in *Proceedings of the Twentieth Annual ACM-SIAM Symposium on Discrete Algorithms*, Vol. 1, 422–431 (Society for Industrial and Applied Mathematics, 2009).

